# Uphill production of dihydrogen by enzymatic oxidation of glucose without an external energy source

**DOI:** 10.1038/s41467-018-05704-5

**Published:** 2018-08-13

**Authors:** Emmanuel Suraniti, Pascal Merzeau, Jérôme Roche, Sébastien Gounel, Andrew G. Mark, Peer Fischer, Nicolas Mano, Alexander Kuhn

**Affiliations:** 1 Max Planck Institute for Intelligent Systems, Heisenbergstr. 3, 70569 Stuttgart, Germany; 2 Centre de Recherche Paul Pascal (CRPP), CNRS UMR 5031, Univ. Bordeaux, 115 Avenue du Docteur Schweitzer, 33600 Pessac, France; 3 CIRIMAT, Université de Toulouse, UPS-INP-CNRS, 118 Route de Narbonne, 31062 Toulouse Cedex 09, France; 4 Institute of Physical Chemistry, University of Stuttgart, Pfaffenwaldring 55, 70569 Stuttgart, Germany; 5 Univ. Bordeaux, CNRS UMR 5255, Bordeaux INP, ENSCBP, 16 avenue Pey-Berland, 33600 Pessac, France

## Abstract

Chemical systems do not allow the coupling of energy from several simple reactions to drive a subsequent reaction, which takes place in the same medium and leads to a product with a higher energy than the one released during the first reaction. Gibbs energy considerations thus are not favorable to drive e.g., water splitting by the direct oxidation of glucose as a model reaction. Here, we show that it is nevertheless possible to carry out such an energetically uphill reaction, if the electrons released in the oxidation reaction are temporarily stored in an electromagnetic system, which is then used to raise the electrons’ potential energy so that they can power the electrolysis of water in a second step. We thereby demonstrate the general concept that lower energy delivering chemical reactions can be used to enable the formation of higher energy consuming reaction products in a closed system.

## Abstract (web summary)

Most chemical reactions proceed downhill without external energy input. Here, authors employ an electronic flyback and a boost converter to store energy and spontaneously drive an uphill reaction. The concept is exhibited by an enzymatic biofuel cell, driving water splitting in a single compartment.

## Introduction

The free enthalpy, or Gibbs energy *G*, determines whether a chemical reaction can proceed spontaneously or whether an external energy input is needed. If the sum of the free enthalpies of a reaction system is positive, then the reaction can normally only proceed if an external energy source is provided. For instance, an external battery can raise the potential of an electron in an electrochemical process, or light can promote an electron in a reaction to a molecular state with a higher energy. However, for the vast majority of reactions, which proceed in a closed system, that means in the same reaction medium, this is not possible. One might therefore ask the very fundamental question whether energies released in an exergonic reaction can be added up to drive the formation of a higher energy endergonic reaction product of a smaller mole fraction in the one and the same medium. While it is straightforward to multiply the work in mechanical systems to raise the energy of a subsystem, this is far from trivial in a closed chemical system. It is therefore necessary to find means to transform energy from a first reaction and then use it to drive a second reaction where a higher energy product is formed. This is obviously possible if the two reactions occur in physically separated reservoirs, like in the case of a battery acting as a first reaction reservoir, driving an electrochemical reaction in a second reservoir. However, combining both reactions in one-and-the-same environment typically leads to a chemical short-circuit, because both electrochemical systems share the potential of the same electrical ground, with the result that thermodynamics forbids such an uphill reaction. For the same reason it is also not possible to connect several electricity delivering cells in series in the same medium in order to add their individual potential differences to obtain a globally higher value.

Here we demonstrate that it is nevertheless possible to drive a chemical reaction with an overall positive free energy *ΔG* > 0 in the same medium, by temporarily storing energy of a first reaction as electromagnetic energy, which is then used to raise the potential of electrons that subsequently participate in a second reaction. Such a concept is crucial if both reactions have to take place in a single medium, and is therefore of general importance from a thermodynamic point of view, but also more specifically for example in all in vivo applications, where both reactions must proceed in the same solution or environment (i.e., in blood). The mechanism we describe herein could allow e.g., the in situ generation of reactive oxygen species (ROS), needed for the regulation of several biological functions in the body^1^, to suppress tumor growth and induce cell death at specific locations^2^, even though their electrochemical synthesis needs rather high potentials^3^. The concept might also be used for autonomous sensing devices or electrolysis cells to locally generate for example specific drug metabolites, by converting a low energy educt molecule to form a higher energy product molecule. In order to illustrate the philosophy of this concept in a proof-of-concept experiment, reaction products such as the ones mentioned above are not suitable, because detection and quantification of all processes should be easy and straight-forward. Therefore, we demonstrate the general idea of the proposed up-conversion with the electroenzymatic generation of hydrogen as a final high energy product, because its formation can be readily tracked (and quantified) in this model system. In particular, we show that it is possible to convert the energy of the electrooxidation of glucose, coupled to the 4-electron oxygen reduction reaction (*ΔG*_1_ = −223 kJ mol^−1^), to power the formation of dihydrogen by electrolysis of water (*ΔG*_2_ = +239 kJ mol^−1^) in the same reaction vessel.

## Results

### Thermodynamic considerations

As illustrated in Supplementary Fig. 1 and explained in Supplementary Note 1, it is impossible to combine several classic electricity delivering cells in series in one single reaction medium in order to amplify the global potential difference, because this leads to an internal short circuit. The same is in principle true for coupling an electricity producing biofuel cell (BFC), based on the conversion of glucose and oxygen, with an electrolyser delivering oxygen and hydrogen. However, the problem can be circumvented by electronically decoupling both electrochemical cells as described in the following.

**Fig. 1:**
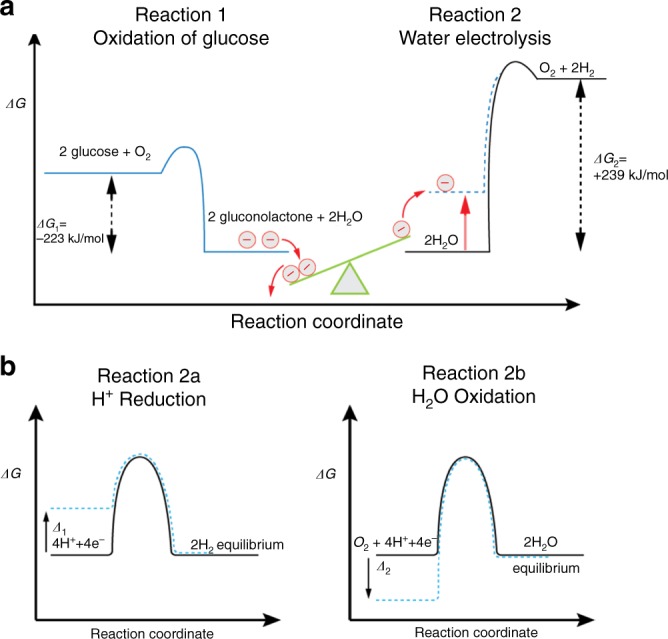
Energetics of the uphill reaction principle. **a** The exergonic glucose oxidation does not provide enough energy to directly drive the electrolysis of water, because the difference in *ΔG* of the two reactions is positive. Using the electrons liberated by the oxidation of multiple glucose molecules allows one to raise the potential of a fraction of these electrons that acquire sufficient energy for the electrolysis of water. **b** The energy provided by reaction 1 is used to promote electrons to a more negative potential (more positive *ΔG*) compared to equilibrium (black line) at the electrolyser cathode by a value *Δ*_1_ (blue dashed line) allowing the proton reduction (reaction 2a) to proceed. On the other hand, the potential at the electrolyser anode (reaction 2b) is changed to a more positive value (more negative *ΔG*) with respect to equilibrium (black line) by a value *Δ*_2_ (blue dashed line). This allows the global reaction 2 to occur because the sum of *Δ*_1_ and *Δ*_2_ is higher than the initially missing 16 kJ mol^−^^1^

The thermodynamics of the considered process is schematically shown in Fig. 1.

The figure illustrates the general concept, because at first sight the spontaneous oxidation of glucose to gluconolactone in order to form gaseous H_2_,

1 $$2\ \mathrm{glucose} \to 2\ \mathrm{gluconolactone} + 2\mathrm{H}_2(\mathrm g)$$

appears to be strictly impossible from a thermodynamic point of view. The evolution of the Gibbs energy *G* governs the sense of chemical reactions performed at constant temperature and pressure. With a positive global *Δ*_*r*_*G* *=*  + 16 kJ mol^−1^, such a reaction can in principle occur only with an external energy input.

Considerable efforts have already been made to explore different hydrogen production pathways by circumventing the thermodynamic restrictions mentioned above. One report uses enzyme cascades to produce hydrogen from deep oxidation of glucose by combining of up to 15 enzymes^4^ under anaerobic conditions^5^. In this case the final product is not gluconolactone but eventually CO_2_ and therefore thermodynamics becomes more favorable. Another interesting approach is based on hydrogenases, however the process has to operate under an inert atmosphere, and sometimes needs elevated temperatures of up to 80 °C in order to achieve adequate efficiencies^6^. It has also been proposed to use microbial electrolysis cells to produce hydrogen^7^. In this case an external input of electricity helps to overcome the mentioned thermodynamic barrier. Finally, methods based on the metabolism of microorganisms, such as fermentation, can also convert glucose into hydrogen, but produce a mixture of CO_2_, H_2_, and eventually methane, which implies purification steps^8^ and activation times^7,9^.

It is therefore an interesting challenge to adapt the here proposed energy up-conversion concept to this model transformation based on a readily available enzyme, working efficiently at low temperatures and under aerobic conditions to produce pure hydrogen as a very visual example of a high energy compound, without the need for any external energy input (e.g., light, heat, electricity, or high energy chemicals). We demonstrate that this is possible by using the concept of intermediate electromagnetic energy storage, combined with an increase in voltage and an electronic decoupling mechanism, allowing us to work in a single reaction medium. An extension of this concept to other thermodynamically unfavorable reaction schemes opens up very interesting perspectives in terms of applications, including in vivo systems, for which, by definition, energy production and energy consumption are intimately coupled and proceed in the same medium.

### Concept of energy upgrading and decoupling

Gibbs energy evolution is directly connected to the spontaneous sense of chemical reactions and, in the particular case of electrochemical reactions, can be expressed by the difference in redox potentials:^10^

2 $$\Delta _rG^0\, = - nF\left( {E_c^0 - E_a^0} \right)$$

where *n* is the number of electrons exchanged between the cathodic (reduction) and anodic (oxidation) sides of an electrochemical cell, and *F* is the Faraday constant (*F* *=* 96,485 C mol^−1^). $E_c^0$ and $E_a^0$ are the standard redox potentials of the cathodic and anodic processes, respectively. Their values are referred to the Standard Hydrogen Electrode, related to the H^+^/H_2_(g) couple at a platinum electrode:

3 $$\rm 4H ^ + + 4e^ - \to 2H_{2({ g})}$$

This half-cell defines the electrochemical potential of 0 V at standard conditions and varies with pH through the Nernst equation, reaching −0.414 V at neutral pH^11^. With a redox potential of −0.345 V at neutral pH^11^, the oxidation of glucose:

4 $$2\ {\rm{glucose} \to 2\ \mathrm{gluconolactone} + 4\mathrm{H}^{+} + 4e^{-}}$$

does not occur spontaneously when it is coupled with the proton reduction reaction. The combination of the two electrochemical processes (Eqs. (3) + (4)) leads to Eq. (1) with a corresponding positive value of Gibbs energy calculated with the formal potentials in Eq. (2).

However, it might be possible to generate H_2_(g) from the electrooxidation of glucose by using the intermittent 4-electron O_2_/H_2_O redox couple:

5 $$\rm O_{2(g)} + 4H^ + + 4e^ - \to 2H_2O$$

With a standard potential of +1.229 V and a formal potential of +0.815 V at neutral pH, the formation of oxygen is not favored when combined with the H^+^/H_2_ redox couple (*Δ*_*r*_*G*^0^ is positive with a value of +239 kJ mol^−1^). Therefore, it is mandatory to inject energy in the form of electrical power to obtain water electrolysis:

6 $$\rm 2H_2O \to O_{2(g)} + 2H_{2(g)}$$

which is the combination of Eqs. (3) and (5) and allows one to deduce a theoretical minimum for the voltage (1.229 V) that needs to be supplied. On the other hand, the oxygen reduction is favored when coupled with the oxidation of glucose (Eq. (4)) with *Δ*_*r*_*G*^0^=−223 kJ mol^−1^ and can spontaneously generate a flow of electrons if the anode and cathode are connected together, giving the global equation:

7 $$\rm 2\ glucose + O_2 \to 2H_2O + 2\ gluconolactone$$

This is the basic operation principle of a glucose/O_2_ BFC. The maximum of theoretical voltage is defined as the electromotive force (emf), $\left( {E_c^0 - E_a^0} \right) = {\textstyle{{{{\Delta }}_rG0} \over {2F}}}$ = 1.16 V. From these values it is obvious that a glucose/O_2_ fuel cell should not be able to drive the electrolysis of water in Eq. (6). This is even more evident when considering that the theoretical value of 1.16 V is at open circuit, that means when no current is drawn. Under experimental conditions with current flow, a more realistic value for the generated potential difference is 0.3 V. Thus it becomes clear that enzymatic BFC are intrinsically low power/voltage devices, and therefore various strategies have been put forward to boost their power output by designing porous materials^12,13^, engineering efficient enzymes^14^ or by coupling BFC to power conversion devices such as the so-called charge pumps or boost-converters^15^. Nevertheless, it has been possible to use such fuel cells for various applications^16–18^, including powering Application Specific Integrated Circuits (ASIC)^19^.

**Fig. 2:**
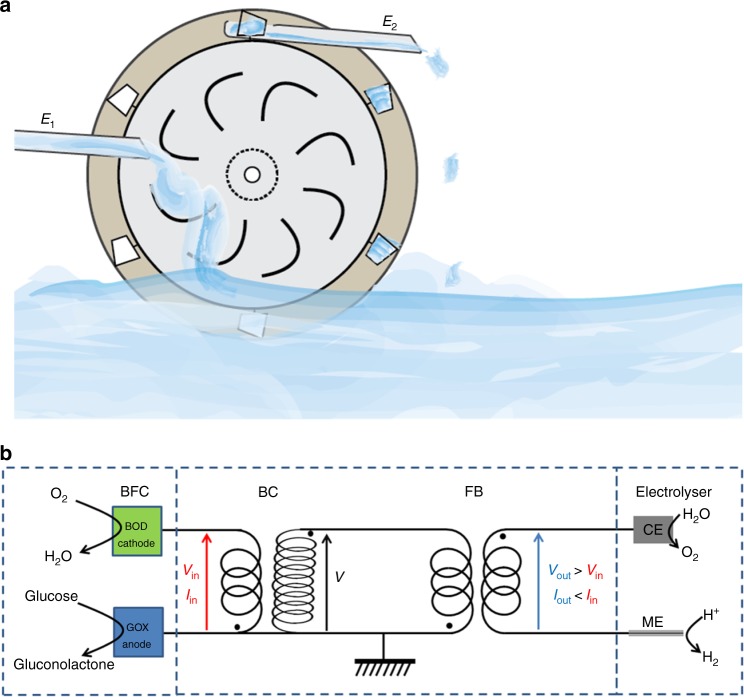
Principle of power conversion. **a** The concept of the uphill reaction scheme can be illustrated by a water wheel. A continuous water flux from the left at an energy level *E*_1_ can turn a paddle wheel, which periodically lifts a smaller water volume to a higher energy level *E*_2_ on the right**. b** This principle is applied to the redox reactions of this work where the energy provided by the oxidation of several glucose molecules in a biofuel cell (BFC) powers an electronic circuit that first raises the potential (voltage) in a boost-converter (BC), followed by a flyback (FB) that electrically isolates the electrolyser (on the right) from the input. The electrolyser is composed of a microelectrode (ME) and a counter electrode (CE). Power conversion and transfer steps are only depicted by transformers, whereas switches and rectifiers are omitted for simplicity. It is important to note that the reaction proceeds in a single vessel and that no external energy is provided. This results in the production of dihydrogen from proton reduction in the same solution as the glucose oxidation, the latter providing the overall driving force

Here we propose such a boost-converter, combined with a decoupling flyback module as a way to produce higher energy electrons, needed for the electrolysis of water, from lower energy ones, provided by a glucose/O_2_ BFC. An analogy of the concept is depicted in Fig. 2a and the electronic equivalent is shown in Fig. 2b as well as in Supplementary Fig. 2 for more details about the boost-converter and flyback. Low energy electrons provided from a first electrochemical system, here illustrated as a water current with a certain gravitational potential (Fig. 2a), are used to raise the energy of electrons needed by a second set of electrochemical reactions, symbolized by the lifting of the smaller water buckets of the watermill. The energy of the electrons is represented by their height. Here the BFC supplies power from the oxidation of glucose at the anode and the simultaneous reduction of oxygen at the cathode to a boost-converter (Fig. 2b). The maximal theoretical voltage of the BFC is 1.16 V, which is clearly below the theoretical minimum voltage of 1.23 V required for the electrolysis of water. In practice an even higher potential of at least 1.7 V is needed for this reaction, due to overpotentials at the electrodes^20^.

The boost-converter transforms the power delivered by the BFC into electromagnetic energy, which in turn is converted back to an output voltage of 3.1 V, schematically represented by the higher level of the water outflow in Fig. 2a. The increase in voltage is balanced by a corresponding decrease in current (the number of water releasing buckets at the outer perimeter is smaller and the water flux less than the input flux on the left), so that the overall energy of the system remains balanced. Such an up-conversion of voltage or charge pump has been already used successfully to power external electronic devices that require a high voltage, despite the modest potential difference that can be harvested from BFC^15,21,22^. However using this strategy for driving another electrochemical conversion (such as water electrolysis or the electrosynthesis of other molecules mentioned in the introduction), taking place in the same reservoir as the BFC, is in principle impossible due to the intrinsic coupling of the two electrochemical systems via the common electrolyte (Supplementary Fig. 1d). This means that if the BFC is placed together with the electrolyser in the same solution, then the oxidation of glucose would have to occur at the same potential, relative to the solution, as the proton reduction, which obviously is not possible. In order to allow two different co-existing potentials in the same reaction medium, the simultaneous operation of the BFC and the electrolyser therefore requires a galvanic separation of the electrodes. This is the most original feature of the presented system. It can be achieved by incorporating a transformer that is internally triggered to charge and discharge the flyback (Supplementary Note 2)^23^. The input energy is periodically transferred from the first to the second winding of the transformer (Supplementary Fig. 2b), with the output coil disconnected from the input ground. The power supplied by the BFC can be transferred to the electrolyser at a different potential, by connecting the boost-converter in series with the flyback. This makes both sub-devices electrically independent and allows operating them in the same medium analogous to what is schematically shown in Fig. 2a by the pulsed water transport and release.

### Comparison of a one-compartment and two-compartment set-up

**Fig. 3:**
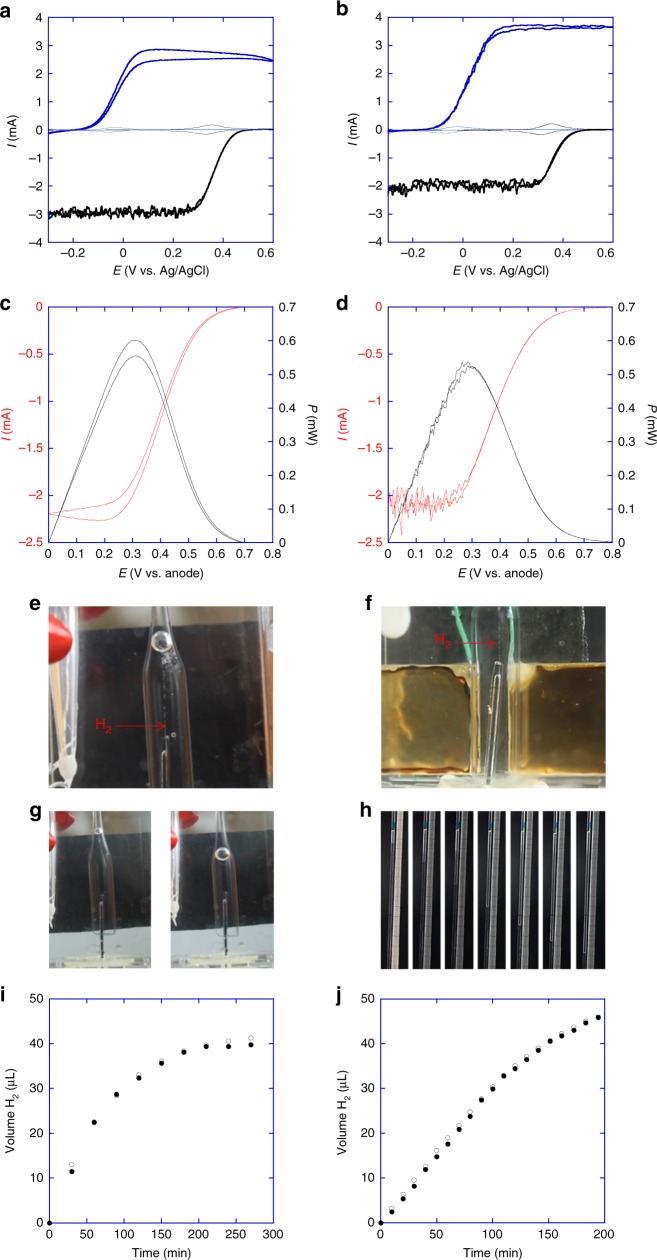
Examples of experiments of H_2_ production powered by glucose/O_2_ biofuel cells. The left column presents an experiment with the electrolyser separated from the BFC compartment, while the right column shows results for the BFC and electrolysis running in the same medium. **a**, **b** Electrochemical characterization at 37 °C of the anode (blue lines) and cathode (black lines) in argon saturated phosphate buffer (thin lines) or in oxygen saturated buffer containing 50 mM of glucose (thick lines). Polarization (red lines) and power (black lines) curves of the biofuel cells for **c** the two-compartment set-up or **d** the one-compartment configuration. **e**, **f** are pictures of the H_2_ generation at the microelectrode in the two and one compartment case respectively. **g**, **h** illustrate the gas collection as a function of time (see Supplementary Movies 1 and 2). For the two-compartment experiment pictures are shown at the start of the reaction and after 4 h. For the one-compartment experiment, pictures of the capillary in which the gas is collected are taken every 30 min. **i**, **j** show the comparison of the H_2_ volume measurement (full circles) and the faradaic estimation (open circles) for the two configurations

In order to demonstrate that switching from a classic two-compartment experiment to the new one-compartment set-up doesn’t induce any substantial losses of power or other disadvantages, we have compared experiments performed with a BFC, which is separated from the electrolyser (Fig. 3 left column), only using a boost-converter as the power conversion system, with a one-compartment configuration (Fig. 3 right column) where the boost-converter was combined with a flyback in order to ensure electrical decoupling.

A first set of experiments was devoted to the optimization of the BFC in order to ensure a maximized power and voltage output as well as a convenient lifetime. BFCs with anodes based on glucose oxidase (GOx) or glucose dehydrogenase (GDH) were designed and their performance with respect to water electrolysis was compared under different experimental conditions. Finally, BFCs with a GOx anode were chosen (see Supplementary Note 3 and Supplementary Fig. 3), with 100 mM phosphate buffer pH 7.2 as the medium. On the electrolysis side, the compatibility of this buffer with hydrogen production was tested and a 50 µm-diameter platinum electrode was preferred to a 100 µm-diameter one (Supplementary Note 3 and Supplementary Fig. 4).

Square planar gold electrodes (2 cm²) are then modified with enzymatic redox hydrogels comprising osmium redox polymers and GOx from Aspergillus Niger^24^ at the anode and bilirubin oxidase (BOD) from *Magnaporthe* *oryzae*^25^ at the cathode. The use of osmium redox polymers as mediators allows connecting electronically the redox centers of enzymes to the electrode surfaces, irrespective of their orientation in a 3D hydrogel matrix permeable to the respective fuel molecules^26^. Fig. 3a, b illustrate the electrochemical characterization of the BFCs providing the driving force. The electrocatalytic activity of the anode (blue curves) and cathode (black curves) is evaluated by cyclic voltammetry at low scan rates (5 mV s^−1^) at 37 °C in the absence (thin line) or presence (thick line) of the enzymatic substrates. In the absence of substrates (in 100 mM phosphate buffer pH 7.2 solution saturated with argon), only the reversible redox waves of the anodic and cathodic mediators can be detected at −0.04 V vs. Ag/AgCl and at +0.33 V vs. Ag/AgCl respectively.

In the presence of 50 mM glucose and in O_2_-saturated buffer, electrocatalysis, exhibiting a typical sigmoidal shape, is observed for both electrodes. The noise in the low potential regime of the oxygen reduction currents is due to the mass-transport limitations in this potential range, which make the current very sensitive to changes in convection caused by the oxygen bubbling. A maximal current of 3.6 mA at the anode and −2.1 mA at the cathode for the one-compartment cell was attained. It was respectively 2.85 mA and −3 mA for the two-compartment experiment. The difference in cathodic current can be explained by the difference in O_2_ convection related to the different cell geometry, while the difference in the anodic currents is within the typical 20% of experimental errors for such systems.

In order to record the polarization curves (red curves in Fig. 3c, d), which allow the characterization of BFCs, the current is measured at the cathode by cyclic voltammetry at 5 mV s^−1^, taking the anode as a reference electrode. These polarization curves allow then plotting the power curve as the major characteristics of the BFC (black curves in Fig. 3c, d).

These polarization and power curves are rather similar when comparing the one and two compartment experiments. Peak powers are 525 and 555 µW respectively, with short-circuit currents of 2.1 and 2.25 mA. The polarization and power curves appear noisier in the one-compartment set-up, because the current is more influenced by mass-transport variations induced by O_2_ bubbling in a set-up with a globally smaller volume (Supplementary Note 4 and Supplementary Fig. 5).

### Hydrogen production

The currents delivered by the two set-ups described above can be directly used for the generation of hydrogen after boosting the voltage. Figure 3e, f shows pictures of the generation of H_2_ at the platinum microelectrode, either located in a separate electrolyser cell, or sharing the cell with the BFC (see also Supplementary Movies 1 and 2). One can quantify the gas volume by redirecting the bubble at regular time intervals into a capillary (two-compartment experiment, Fig. 3g) or via regular imaging of the capillary in which the gas is constantly collected (Fig. 3h). These data were compared with the amount of H_2_ expected from the electric charge consumed by the electrolyser. A very good agreement was obtained, with a faradaic efficiency of the electrolyser close to 100% (Fig. 3i, j).

Initial rates of H_2_ production are in the present examples 22.5 µL h^−1^ for the two-compartment experiment and 17.6 µL h^−1^ for the one-compartment set-up. This slight decrease is essentially due to the additional energy needed for driving the flyback (only present in the one compartment experiment) in order to allow electric decoupling of the fuel cell electrodes from the electrolyser electrodes (see efficiency calculations in Supplementary Note 5). However, taking into account the different electrolyte volumes in the experimental set-ups, the volume production rates are slightly in favor of the one-compartment device (11.5 µmol h^−1^ L^−1^ for the two-compartment set-up and 17.3 µmol h^−1^ L^−1^ for the one-compartment experiment). This illustrates that further miniaturization of the one-compartment system, which is rather difficult for the two-compartment system, could lead to an even higher volumetric production rate of H_2_.

The reaction in the one-compartment configuration has been followed for 200 min, and during this time the produced hydrogen gas is collected (Fig. 3h). The boost-converter operates optimally during the first 80 min. During this period, the BFC provides enough power for the boost-converter to keep a 3.1 V output and a stable output current of 57 µA, ultimately supplying constant power to the electrolyser (Supplementary Note 5 and Supplementary Fig. 6a). After that, the slow decrease in BFC output potential reaches a point where optimal operation of the boost-converter cannot be maintained, resulting in a gradual decrease in power at all stages of the electronic conversion (Supplementary Fig. 6b). The system can still operate, however with somewhat smaller currents at the electrolyser. The decrease in power density of the BFC may be attributed to the gradual deactivation of the anodic enzyme due to a concomitant production of small amounts of hydrogen peroxide.

## Discussion

These experiments demonstrate that the set-up allows the two couples of electrochemical reactions to proceed independently in the same medium, despite the fact that the global reaction is unfavorable from a thermodynamic point of view (Fig. 1). The main novelty of the system lies in the synergetic combination of the electrochemical components with the flyback/boost-converter device. Flyback and boost-converter units have been already employed e.g. in combination with microbial fuel cells (MFC), however none of the studies report simultaneous up-conversion of the power and galvanic isolation from a second electrochemical system operating in the same solution. For example a flyback has been used to increase the energy harvesting performance, but the energy was stored in an output capacitor and has not been reinjected into the system to operate an additional integrated electrochemical cell^23^. Similar approaches allowed the power management between a MFCs and an external electronic element as a load^27^, or the energy harvesting from several MFCs connected in series^28^, but the final power output was not fed back into the same system to drive an independent electrochemical reaction. In the present case, the simultaneous increase and decoupling of the voltage enables the electrolysis of water by changing the potentials of the individual electrolyser electrodes, driven by the thermodynamically unfavorable glucose oxidation (Fig. 2b). More precisely, electrons produced by the oxidation of glucose are in principle available for the reduction of protons, although their initial energy is too low for triggering this transformation. This can be circumvented by promoting artificially these electrons to a higher energy level; however, it requires a decrease of the current available for the electrolysis. It follows that more oxygen is consumed by the BFC than produced by the electrolyser. The difference between consumption and production of oxygen as an intermittent species corresponds to the voltage up-conversion and the charge consumed for its operation, as well as for the powering of the decoupling flyback circuit. The overall stoichiometry of the reactions involved in the BFC and the electrolyser is therefore modified by the power conversion step. H_2_(g) is effectively produced by glucose oxidation, however the process is no longer described by Eq. 1 in terms of quantitative stoichiometry.

With two electrons involved in both the oxidation of one glucose molecule and reduction of two H^+^ to form one H_2_, the conversion efficiency of glucose into dihydrogen can simply be estimated from the ratio of charges at the input (BFC) and the output (electrolyser) of the system. During the reaction time of 200 min a total of 46 µl of H_2_, corresponding to 1.81 µmol, is generated from the consumption of 35.4 µmol glucose. Thus, the glucose to H_2_ conversion yield is 5.1% for the one-compartment set-up, referring to the ratio of the total input charge of 6.84 C and an output of 0.35 C. In comparison, the total yield of hydrogen production for the two-compartment device is 6.5% (with 5.06 and 0.33 C at the input and output respectively). This difference is due to the internal energy consumption by the flyback module, which has an efficiency of 65% (64.9 ± 2% over three experiments). Combined with average efficiencies of 70% for the boost-converter, the total system has a power conversion efficiency that varies between 45 and 48% (see detailed efficiency calculations in Supplementary Note 5 and Supplementary Fig. 7-9).

With 0.051 mol of H_2_ produced per mol of glucose consumed, the efficiency of the process presented here is very similar to the state of the art of photobioelectrochemical systems in terms of yield^29^. Concerning the production rate (mmol(H_2_) L^−1^ h^−1^), a direct quantitative comparison with literature values is not straightforward as the cell volume or the quantity of enzymes are sometimes not stated or cannot easily be converted to meaningful values with respect to the concentration of immobilized enzymes in the present case. In the proof-of-concept experiment reported here, hydrogen is generated at a rate of 17.3 µmol(H_2_) L^−1^ h^−1^, but the system has been operated only with a microelectrode for the hydrogen production. As in the present experiments the BFC was not working in the transport-limited regime (neither for O_2_ nor for glucose) it is obvious, that when further increasing the geometric size of the electrodes, or using porous electrodes instead of flat electrodes, one can potentially increase the hydrogen production rate. We could show in the past that using macroporous electrodes allows increasing the oxygen reduction current by up to one order of magnitude^30^. This strategy can be adapted in order to obtain a perfect match between anodic and cathodic currents and increase the maximum power generated by the BFC. We calculated for example that when using larger porous electrodes with 15 half layers of 600 nm pores^31,32^ it is possible to reach production rates of the order of mmol(H_2_) L^−1^ h^−1^, which are comparable with literature values^33^. In the present study we used flat electrodes for the BFC with a larger surface area compared to the microelectrode in the electrolyser. The size ratio of the electrode surfaces has been chosen because it allows the power of the BFC to be concentrated on smaller electrolyzer electrodes in such a way that the resulting current density on the microelectrode is high enough to produce macroscopic hydrogen bubbles for better visualization of the process. Bigger electrolyzer electrodes would also produce hydrogen, but with a lower gas density at the electrode surface, which would lead to almost invisible bubbles or a continuous dissolution of the produced gas in the electrolyte because locally the gas saturation threshold isn’t reached.

Even though the chemical reactions chosen for the present work serve just as a model system, it is quite important that the H_2_ production powered by the glucose/O_2_ BFC can be carried out in one single medium, thereby imitating the enzymatic production of H_2_ using glucose as a substrate. One further positive aspect of this scheme is that GOx is a robust enzyme that can be employed to generate dihydrogen, a clear advantage given that most of the hydrogenases used for the enzymatic production of hydrogen reported to date are much less stable^34^. The immobilization of the enzymes on the electrode surfaces together with a redox polymer not only yields high selectivity, but most importantly does not require the use of seals, containers or membranes. Combined with the electronic decoupling of the power generation from the electrolysis, the presented scheme can readily be miniaturized, and only needs immersion in a glucose containing solution in order to produce significant amounts of pure hydrogen. The device can be therefore considered as an artificial version of a hydrogenase, but with the advantage of operating under ambient aerobic conditions and with higher stability.

Beyond efficiency and practical engineering aspects, the impact of these results is much more general because they demonstrate how lower energy producing chemical reactions can be used to enable the formation of higher energy consuming reaction products that are otherwise thermodynamically forbidden in a closed system, but are generally more valuable. The concept relies on chemical energy that can be transformed intermittently into electromagnetic energy and thus allows decoupling of two (electro)chemical systems. This leads to the very general possibility of combining a large set of electron transfer reactions, no matter whether they are globally down-hill or up-hill from an energetic point of view. For the up-hill reactions, the voltage, needed to increase the energy of a participating electron, is obtained by decreasing the electric current, thus conserving the overall energy. Based on this concept, any electrochemical fuel cell, and in particular those using abundant and cheap reactants, can thus be employed as a power source for other electrochemical reactions operating at a higher electrode potential. Electrically uncoupling the two devices opens the door to an almost unlimited number of combinations of electricity producing and electricity consuming redox systems in the same reaction medium, which might be of particular relevance for in vivo systems where both, by definition, are located in the same solution. Another example might be corrosion prevention where a low energy producing reaction, occurring in the surrounding medium, constitutes, after voltage up-conversion, the power source for applying the necessary potential to the metal which needs to be protected in the same medium.

## Methods

### Chemicals and materials

All aqueous solutions were prepared in Milli-Q water (18.2 MΩ cm). All reagents were of analytical grade. D^+^-Glucose 99.6 %, mono-basic and di-basic sodium phosphate, and H_2_SO_4_ 99% were purchased from Sigma. GOx from Aspergillus Niger^24^ and BOD from *Magnaporthe* *oryzae*^25^ were produced as previously described. Anodic and cathodic redox polymers were also synthesized according to literature procedures^35,36^.

All electrochemical measurements, essentially for characterization of the BFC electrodes, were performed with bi-potentiostats from CH Instruments (842b or 760C), with Ag/AgCl (KCl 3 M) and platinum wires from BAS as reference and counter electrode.

### BFC preparation

2 cm² gold electrodes were prepared by sputtering. The gold electrodes were fixed on a T-shaped glass support with silicon paste (CAF4). After cleaning they were checked with cyclic voltammetry in H_2_SO_4_ 0.1 M (series of 50 CVs from −0.2 to +1.6 V vs. Ag/AgCl, 100 mV s^−1^). After rinsing and drying, hydrogels were deposited on the gold surfaces.

43.3 µl of anodic redox polymer (PVP-Os[(1,1-dimethyl-2,2′biimidazole)2-2-[6 methylpyrid- 2yl] imidazole]^2+/3+^; 7.14 mg mL^−1^ in water), 39.4 µL of GOx (5 mg mL^−1^ in water), and 28.1 µL of polyethyleneglycol diglycidyl ether (PEGDGE; Polysciences Inc., Warrington, PA, USA, 2 mg mL^−1^ in water) were mixed to obtain a 55/35/10 wt% solution, from which 110.8 µL were spread on a freshly cleaned electrode (the anode of the BFC) for a total hydrogel loading of 250 µg cm^−2^ ^37^. 35.2 µL of cathodic redox polymer (PAA-PVI-[Os(4,4′-dichloro-2,2′-bipyridine)2Cl]^+/2+^, 10 mg mL^−1^ in water), 33.8 µL of BOD from *Magnaporthe* *oryzae* (5 mg mL^−1^ in water), and 20.9 µL of PEGDGE (2 mg mL^−1^ in water) were mixed to obtain a 30/62.6/7.4 wt% mixture, and 89.8 µL were deposited on a second gold electrode (the cathode of the BFC) for a total loading of 250 µg cm^−2^ ^35^. The electrodes were kept 42 h at 4 °C, covered, and taken out of the fridge 45 min before the first electrochemical characterization.

### Platinum microelectrodes

Microelectrodes were prepared from a 50 or 100 µm-diameter platinum wire (from Goodfellow) and a borosilicate glass capillary (World Precision Instrument) using a laser puller (Sutter Instrument P2000), following a literature procedure^38^. The connection was ensured with carbon powder (HS40) and Araldite glue was used to seal the electrode. Their diameter was checked by electron microscopy (Hitachi TM-1000) and by cyclic voltammetry at low scan rates (2 mV s^−1^) in Ru(NH_3_)_6_Cl_3_ 5 mM in PBS. Prior to the H_2_ production experiments, the microelectrode was polished on fine grain paper, and cleaned with two series of 50 cyclic voltamogramms at 100 mV s^−1^ in H_2_SO_4_ 0.1 M saturated with argon. A chronoamperometry at −3.1 V vs. a platinum counter electrode was then performed in buffer until regular and constant hydrogen production could be observed at the microelectrode.

### Electrochemical measurements

A standard three electrode configuration was used to characterize the anode and cathode of the BFC in 100 mM phosphate buffer at pH 7.2 at 37 °C in a temperature-stabilized cell. Cyclic voltammetry at 100 and 5 mV s^−1^ was first performed for the BFC in the absence of glucose and in argon-purged buffer, taking Ag/AgCl as a reference electrode. The electrocatalysis of the BFC electrodes as well as polarization curves were recorded in a home-made thermostated cell (see Supplementary Methods and Supplementary Fig. 10a). The CVs were performed at 5 mV s^−1^ in O_2_-saturated buffer with 50 mM glucose. For tracing the BFC polarization curves, reference and counter electrode connections of the potentiostat were connected to the anode in order to measure the current as a function of the potential difference between the two electrodes. The power curve is obtained from the polarization measurement after multiplying the current by the voltage of the BFC.

### Power conversion

An ultra-low power boost-converter on demo board BQ25504 EVM from Texas Instrument was used for power conversion of the BFC. The BFC was connected to the input of the boost-converter with the anode as the electrical ground, keeping the boost-converter output open to allow a so-called cold-start during which the capacitance reaches a voltage of 3.1 V.

As the electrical ground is common to the input and the output in this commercial device, the operation of a BFC in the same solution as the one of the electrolyser requires decoupling of the two grounds. This is achieved with a home-made flyback module described in the Supplementary Note 2 (scheme in Supplementary Fig. 2b). The flyback was just used for galvanic isolation and the boost converter to raise the output voltage of the BFC. This combination has been employed because the switching MOSFET at the output of the primary winding of the flyback requires a tension above the one that the BFC can deliver. Therefore, it is not possible to place the flyback directly at the output of the BFC without the intermediate booster. The electrolyser is connected to the output of the flyback, and as soon as the cold-start is completed, the output of the boost-converter is connected to the flyback’s input.

### Electrolysis cell

A platinum microelectrode is introduced in the electrolysis cell, and covered by a H_2_ collector (for details see Supplementary Methods and Supplementary Fig. 10b). The experiments were conducted in a 50 mM glucose solution with oxygen saturation (bubbling) at 37 °C. When necessary 20 nM catalase was also added to limit the BFC degradation caused by the side-production of H_2_O_2_ at the anode^39^. The H_2_ collector was filled with the buffer solution and a 5 µL air bubble to permit the measurement of the H_2_ gas evolution (see Supplementary Fig. 10b and 10c).

### Potential/current and gas volume measurements

The voltage and current is determined at the input of the boost-converter (corresponding to the output of the BFC) with a National Instrument NI USB-6211 16 bits 16-channels tension measurement module, with an additional resistance in parallel for measuring the current. The current and tension between the boost-converter and the flyback are respectively measured with a Keithley 2000 multimeter and the NI USB-6211. The independent voltages of the microelectrode and the platinum counter electrode in the electrolyser are also measured and referred to the common GND of the anode of the BFC. The hydrogen evolution is monitored with a full-HD camera (Logitech C930E).

### Data availability

All relevant data that support the current findings and which are not included in the main manuscript or the Supplementary Information are available from the corresponding author on request.

## Electronic supplementary material

Supplementary Information

Description of Additional Supplementary Files

Supplementary Movie 1

Supplementary Movie 2
