# Supplementary material for: Uphill production of dihydrogen by enzymatic oxidation of glucose without an external energy source
Source: Nat Commun. 2018 Aug 13;9:3229. doi: 10.1038/s41467-018-05704-5 (PMC6089969; doi:10.1038/s41467-018-05704-5)
Supplement: Supplementary file 1 — Supplementary Information [file 41467_2018_5704_MOESM1_ESM.pdf]

Supplementary Information

Uphill production of dihydrogen by enzymatic oxidation of glucose without an external energy source

Suraniti et al.

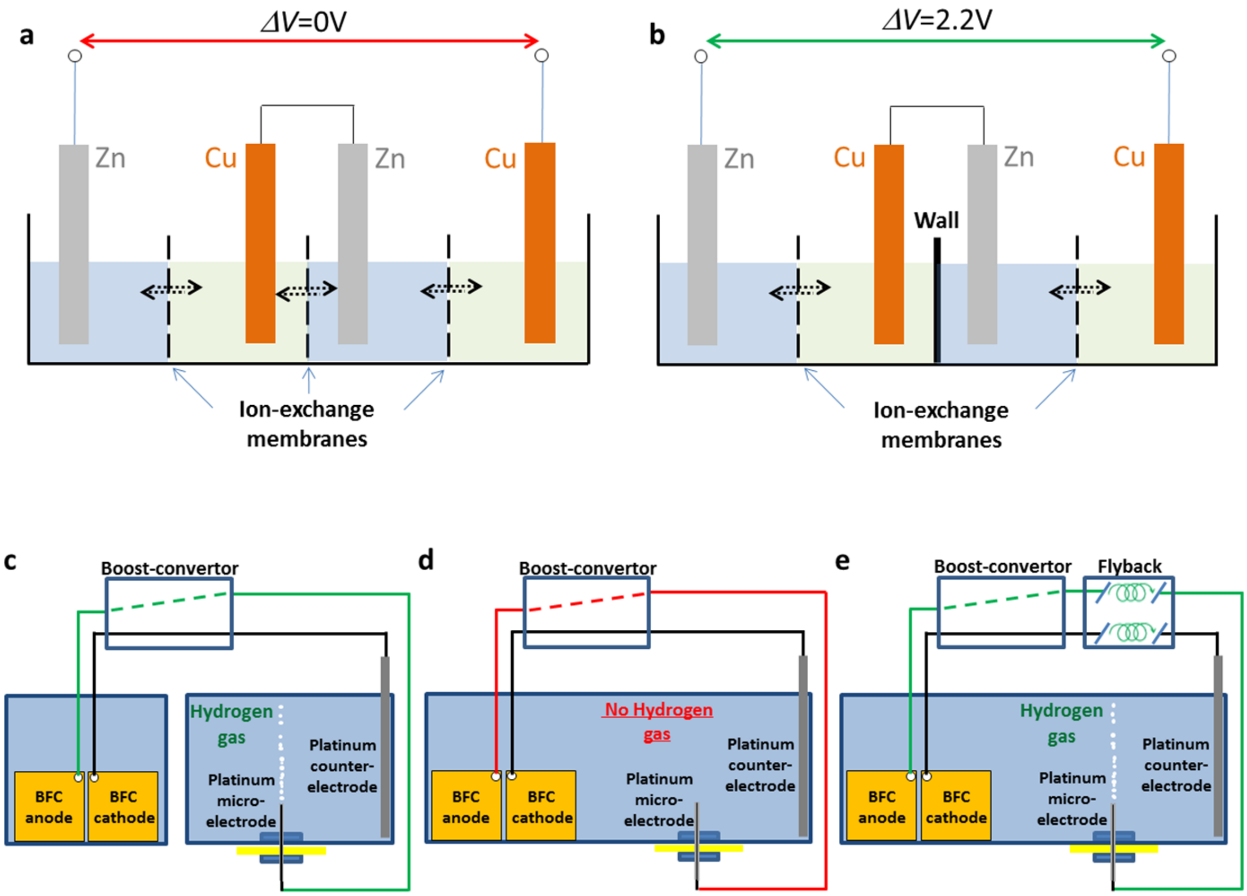

Supplementary Figure 1. **Influence of compartmentalization on the coupling of electrochemical cells. a** Scheme of two Daniell cells connected in series immersed in the same electrolyte solutions. **b** Two Daniell cells connected in series but in separate solutions. **c** Scheme of a BFC and electrolyser in separate electrolytes connected via a boost-converter. **d** Scheme of a BFC and electrolyser sharing the same medium connected via a boost-converter. **e** BFC and electrolyser sharing the same electrolyte but connected via the combination of a boost-converter and a flyback.

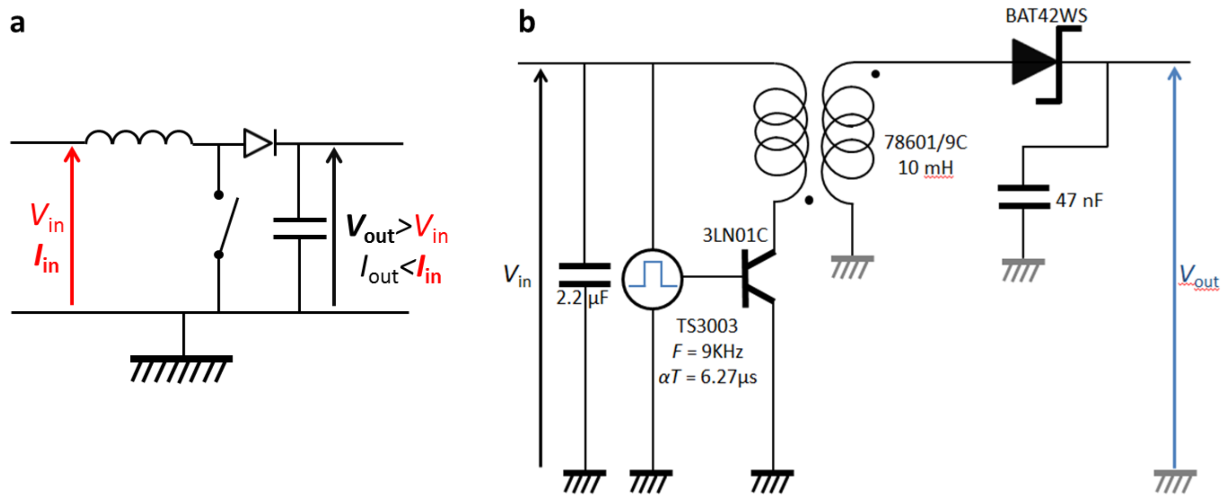

Supplementary Figure 2. Elements of electronics for the voltage up-conversion and galvanic isolation. a General scheme of a boost-converter. Please see the BQ25504 technical information for further details. **b** Scheme and specific settings of the flyback module for decoupling of the electrical grounds.

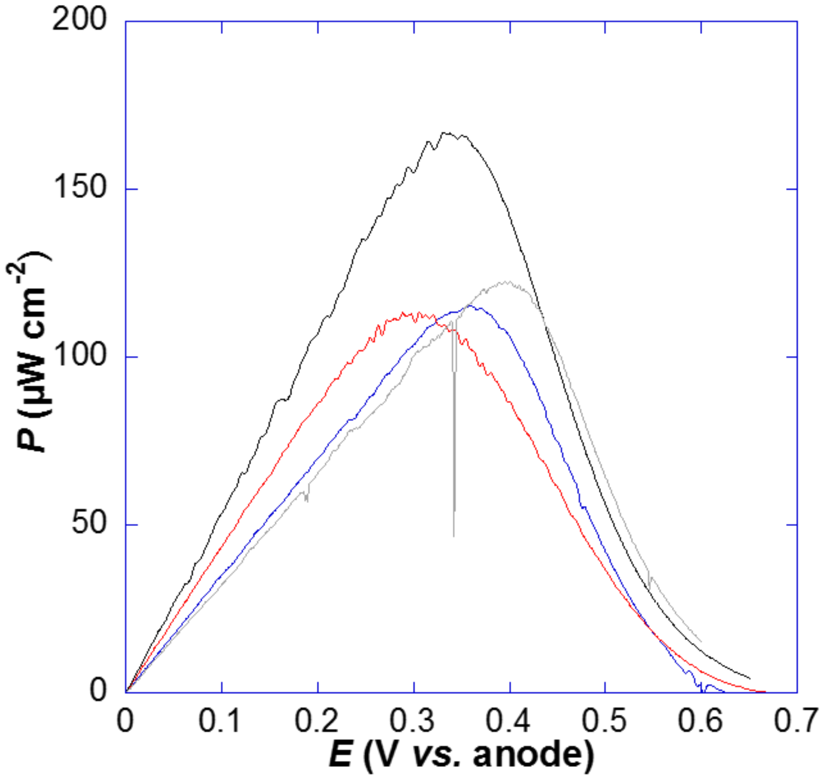

Supplementary Figure 3. Comparison of different anodic enzymes and experimental buffers. Power densities of biofuel cells based on 5mm-square gold electrodes, with a loading of 200 µg cm^{-2} and BOD from *Magnaporthe Oryzae* as the cathodic enzyme, recorded in different buffers at pH 7.2. The anodic enzyme and buffers are: GOx in 100 mM phosphate buffer (black line), GOx in 20 mM phosphate buffer + 140 mM NaCl (grey line), GDH in 100 mM phosphate buffer 100 mM (blue line), GDH in 20 mM Pipes buffer + 3 mM $CaCl_2$ (red line).

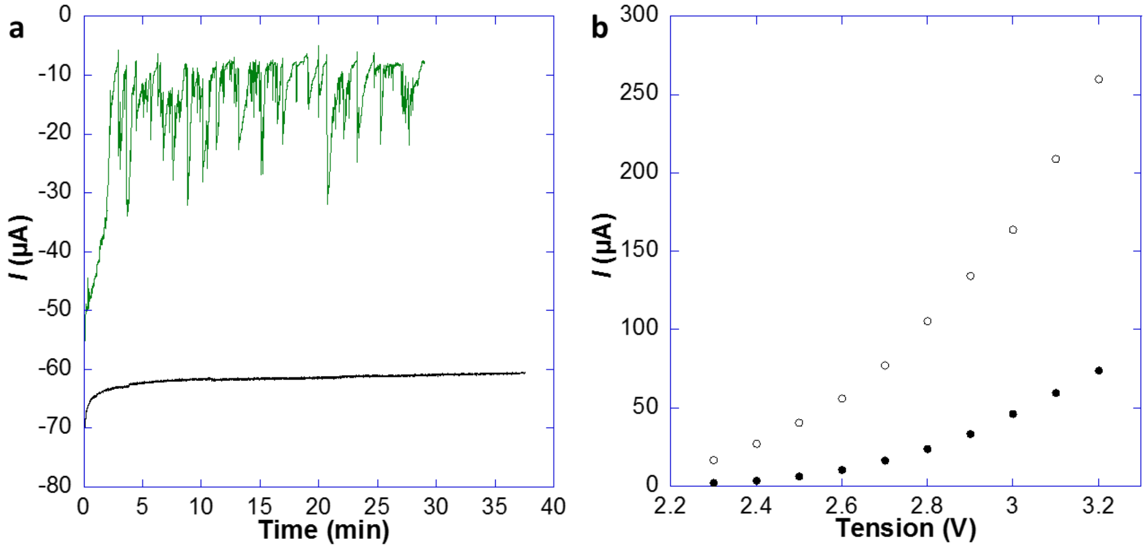

Supplementary Figure 4. Choice of the electrolysis conditions. a The current is followed over time for a 50 µm-diameter platinum electrode set at -3.1 V vs. the platinum counter electrode in phosphate buffer 100 mM pH 7.2 (black line) and in Pipes 20 mM $CaCl_2$ 3 mM pH 7.2 (green line) at 25 °C. **b** The electrolysis current is measured for different tensions of electrolysis for a 100 µm-diameter (open circles) and a 50 µm-diameter (full circles) platinum electrode in phosphate buffer 100 mM pH 7.2 at 25 °C.

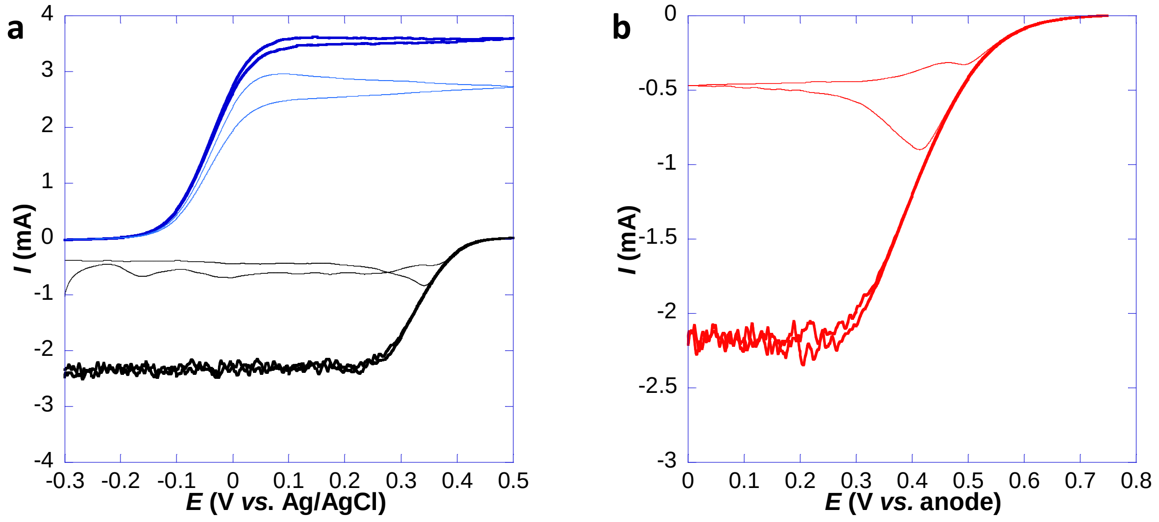

Supplementary Figure 5. Influence of mass-transport on biofuel cell performances. a Electrochemical characterization of the biofuel cell electrodes (anodes in blue, cathodes in black). Cyclic voltammogramms are performed at 5 mV s^{-1} in phosphate buffer 100 mM at pH 7.2 and at 37 °C in the presence of glucose 50 mM and active O_2-bubbling for electrocatalysis (thick lines) or with the oxygen flux just above the solution (thin lines). **b** Polarization curves for the same BFC with O_2-bubbling conditions of **a**.

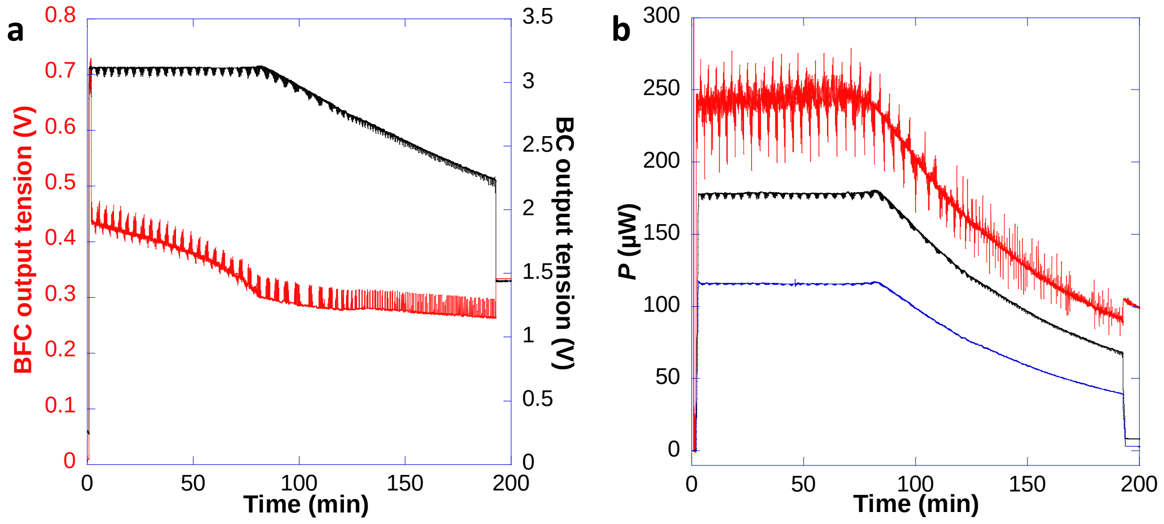

Supplementary Figure 6. Electrical measurements of a one-compartment experiment. a Evolution of BFC output tension (= boost-converter input, red line) and boost-converter output tension (black line) as a function of time. **b** Power evolution as a function of time at the output of the BFC (input of boost-converter, red line), at the output of the boost-converter (input of flyback, black line) and at the output of the flyback (input of electrolyser, blue line). Experiments carried out at 37°C with 50mM glucose solution.

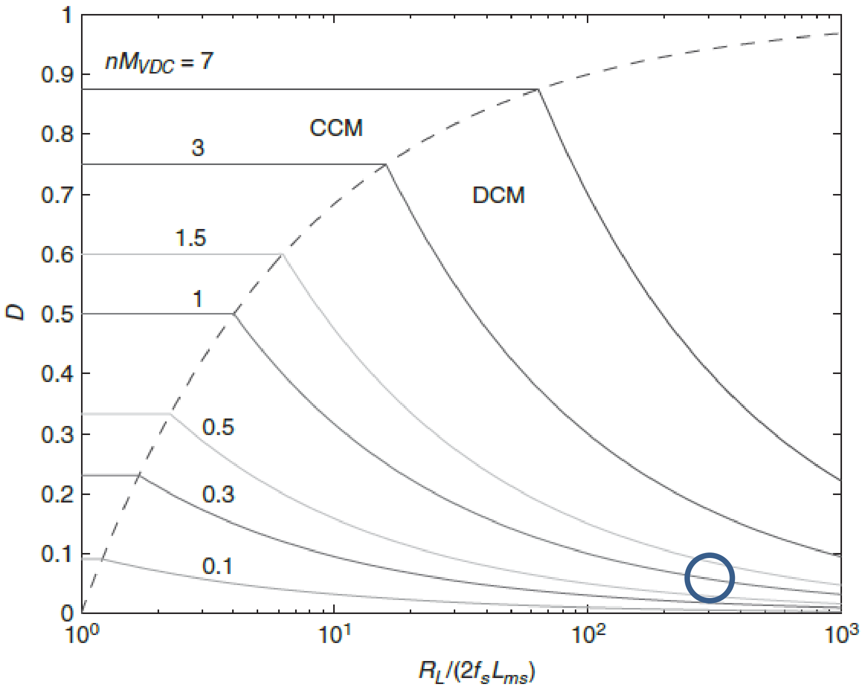

Supplementary Figure 7. Determination of the DCM regime. Duty cycle D as a function of the load resistance $R_L/(2f_sL_{ms})$ at fixed values of $M_{V\ DC}$ for the lossless flyback converter in CCM and DCM. Adapted from [1] with permission from John Wiley & Sons.

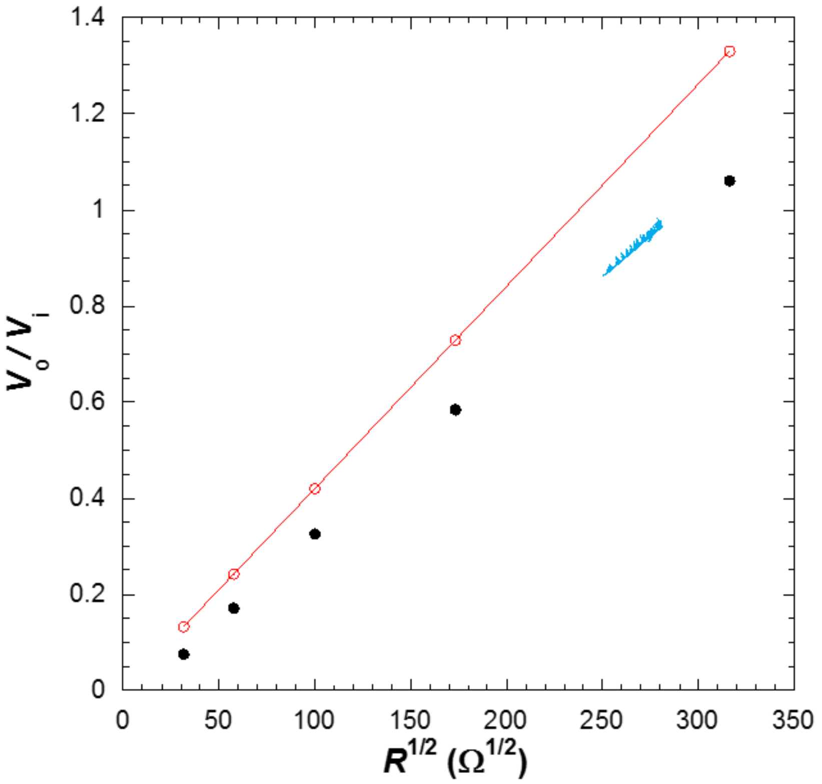

Supplementary Figure 8. Transfer function of the flyback. Transfer function of the flyback for different load resistances. Red dots are the theoretical voltage transfer values whereas black dots indicate the experimental results obtained for well-defined resistors. Small blue points are the data of a typical experiment for the one-compartment production of H_2 powered by the BFC.

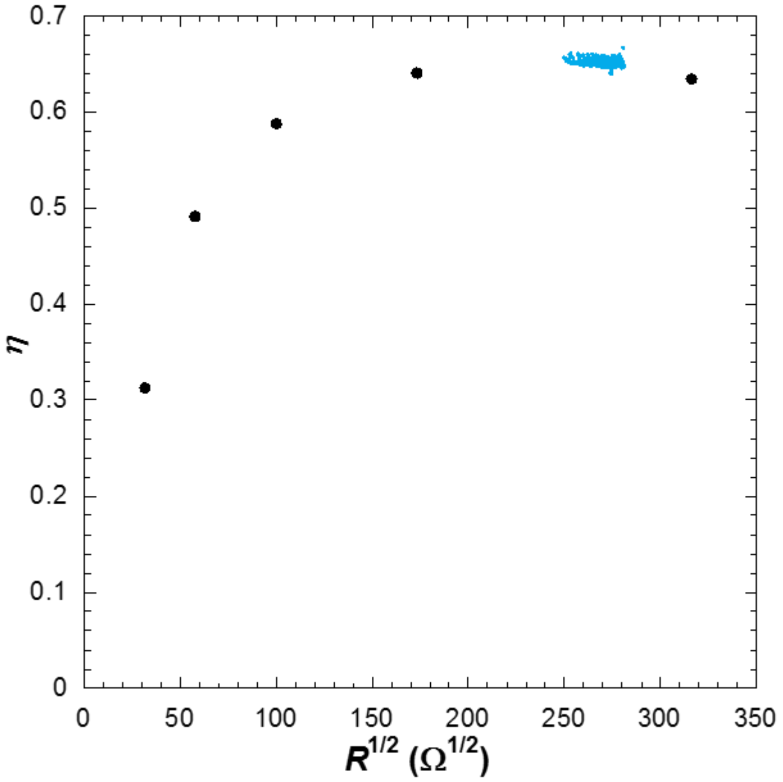

Supplementary Figure 9. Flyback efficiencies. Plot of the measured flyback efficiencies for different load resistances (black points) and the electrolyser as a load (small blue points).

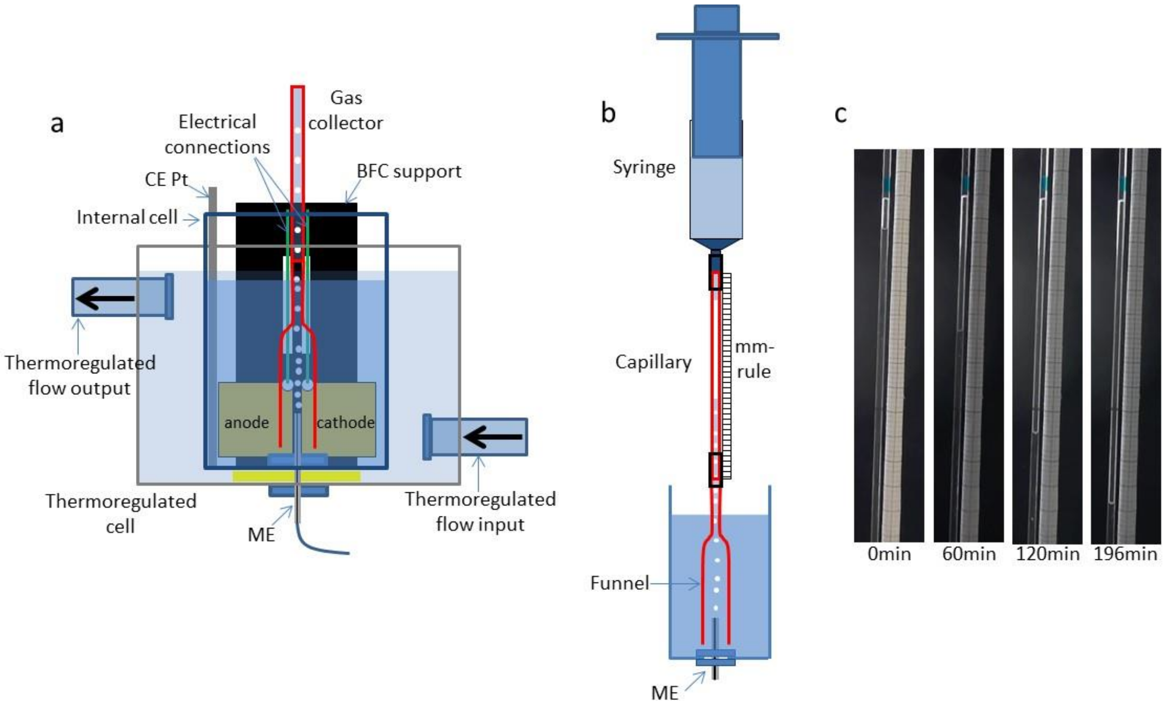

Supplementary Figure 10. Specific experimental set-ups for the operation of electrolysis and biofuel cell in the same compartment and collection of H_2. a Scheme of the single compartment combining the biofuel cell (BFC, composed of an anode and a cathode) and the electrolysis cell composed of a platinum counter electrode (CE Pt) and a platinum microelectrode (ME). **b** Scheme of the gas collector, the volume of H_2 being measured by the length of the capillary filled with gas. **c** Pictures of H_2 collection: at t = 0min only the initial air bubble is present; after 60, 120 and 196 min more and more H_2 is accumulating in the capillary.

Supplementary Notes

Supplemental Note #1 Coupling of electrochemical cells.

The coupling of electrochemical cells in one single compartment usually leads to an internal short-circuit due to the common electric ground shared by several electrodes. This can be exemplified by the serial connection of two classic Daniell cells (Supplementary Figure 1). When the two cells (individual thermodynamic potential difference of 1.1 V under standard conditions) are communicating via the electrolyte, either by a salt bridge or a membrane, no power output is measured (Supplementary Figure 1a). However, separating them into two completely independent cells allow the voltages to add up to a global potential difference of 2.2 V (Supplementary Figure 1b).

An analogous situation is encountered in the present case when the electricity producing biofuel cell is coupled to an electrolyser (Supplementary Figure 1c-e). Physical separation of both units allows the electrolysis to be driven with the power of the biofuel cell after an up-conversion of the voltage via a boost-converter (Supplementary Figure 1c). Combining them in the same electrolyte medium leads to a failure of the system and no electrolysis takes place (Supplementary Fig. 1d). Only the addition of a so-called flyback module permits an electric decoupling of the devices and enables water electrolysis (Supplementary Figure 1e).

Supplemental Note #2 Power conversion and electrical decoupling of BFC and electrolyser.

Power conversion was performed by a boost-converter BQ25504 from Texas Instrument. A general scheme of boost-converters is depicted in Supplementary Figure 2a (please see the manufacturer’s information for the exact scheme of the commercial device). Power conversion is based on the response to changes in current at an inductor, combined

with storage and release of energy by induction/destruction of a magnetic field. When the switch is closed and the current from a generator (the biofuel cell in our case) flows through the inductor directly to the ground (GND), energy is stored. When the switch is open, the current passing through the inductor tends to become lower due to a higher output impedance: the inductor releases energy to maintain the current and its polarity reverses. In this case voltage is added to the voltage of the generator, leading to an output tension of the boost-converter higher than its input.

Supplementary Figure 2b describes the electrical scheme of the flyback designed for this study. The output of the BQ25504 boost-converter constitutes the input of a TS3003 oscillator/timer from Silicon Lab as well as the positive pole of an electrical transformer (78601/9C, 10 mH). The TS3003 triggers the current in the first winding with the help of a MOSFET transistor (3NL01C) placed at its output. Energy is then stored in the magnetic core of the transformer, because a Schottky diode (BAT42WS), situated at the output of the second winding, prohibits the direct transfer. When the first winding circuit is opened by the TS3003, the energy flows in the second winding as the voltage is now of the appropriate sign for the diode to become conductive. Galvanic isolation is thus obtained by energy transfer between the two windings which are connected to independent electrical grounds. The original resistance and capacitor of the TS3003 were replaced by 12 MΩ and 27 pF equivalents to obtain a sampling frequency of 9 kHz and αT of 6.27 µs. These parameters were determined from independent experiments in which the electrolyser was fed through the flyback system by a function generator delivering a 3.1 V amplitude square wave. It was found that this frequency value and square wave width is a good compromise to maintain good power efficiency of around 65 % (see efficiency calculations further down), either at optimal or lower performance of the BFC corresponding to boost-converter output voltages ranging from

2.2 to 3.1 V. Below 2.2 V the flyback efficiency drops dramatically due a tension cut-off effect.

Supplemental Note #3 Preliminary study of the biofuel cell composition and compatibility with electrolysis.

BFCs with anodes based on glucose oxidase (GOx) or glucose dehydrogenase (GDH) were designed on 5 mm-square gold electrodes. The power of the BFCs resulting from their combination with a cathode based on bilirubin oxidase (BOD) from *Magnaporthe Oryzae* were compared in various experimental buffers, corresponding to optimal buffers of the three enzymes (Phosphate buffer 20 mM + NaCl 140 mM for glucose oxidase, Pipes 20 mM with $CaCl_2$ 3 mM at pH 7.2 for GDH, phosphate buffer 100 mM pH 7.2 for BOD). Polarization curves of the BFCs are performed under O_2 bubbling at 5mV s^{-1} and at 37 °C until the power of the BFC reaches stabilization, resulting in the power curves shown in Supplementary Figure 3.

BFCs based on GDH exhibit power densities in the range of 115 µW cm^{-2}, either running in phosphate buffer 100 mM or in Pipes-$CaCl_2$. The slightly lower power value in phosphate buffer (blue curve) is due to the complexation between the phosphate and the calcium, which binds the active site of the enzyme. This is the reason why 50 % of the power is lost after only 6 polarization cycles. In Pipes-$CaCl_2$ (red curve) the cause of the low power is the low activity of BOD in this medium. The same reason explains the similar power of ~120 µW cm^{-2} obtained for a BFC based on a GOx anode and running in its preferential phosphate buffer 20 mM NaCl 140 mM (grey curve). Finally, the GOx BFC running in phosphate buffer 100 mM gave the highest power density, with ~165 µW cm^{-2} (black curve).

Half-lives of these systems under expected conditions of BFC operation for the H_2 production experiments were determined by chronoamperometry by following the current at the cathode over time while imposing a potential difference of +0.3 V *vs.* the anode. The GOx-based BFC possesses the second longest half-life (70 minutes) after the GDH-based BFC running in Pipes-$CaCl_2$ (> 120 minutes). As this longer lifetime could eventually balance the lower power observed for the GDH-based BFC, the water electrolysis was studied in Pipes-$CaCl_2$ by poising the platinum microelectrode at -3.1 V *vs.* the platinum counter electrode. It was systematically observed that in this buffer, the electrolysis drops after only a few minutes (Supplementary Figure 4a). This was ascribed to the formation of solid calcium salt at the microelectrode surface blocking a major part of its activity.

As it exhibited the highest power density, and didn't show any inhibition of the electrolysis in phosphate buffer 100 mM, the BFC based on GOx running in phosphate buffer was selected for our experiments with an expected power in the range of 500 to 600 µW for a BFC with 2 cm² square gold electrodes. Indeed, a power of 0.53 ± 0.1 mW was measured for such biofuel cells with a loading of 200 µg cm^{-2}. BFCs with a hydrogel loading of 250 µg cm^{-2} exhibited a power of 0.55 ± 0.05 mW, indicating that the usual electrocatalysis plateau for BFCs is reached for loadings around 250 µg cm^{-2}.

Considering the 46-48 % power efficiency of the combined boost-converter/flyback system (see efficiency calculation in Supplementary Note 5), crucial for powering the electrolysis by the BFC in the same medium, one can expect a power available for electrolysis of around 245 µW. As the expected tensions of electrolysis are in the range of 3.1 V, corresponding to the boost-converter output voltage, the current used for electrolysis ideally should not exceed 80 µA for an optimal utilization of the system. Supplementary Figure 4b shows that a 100 µm-diameter platinum electrode would consume too much current if powered by a BFC based on flat 2 cm² electrodes, as electrolysis at 3.1 V triggers a current of

more than 200 µA. A 50 µm-diameter electrode would consume only some 185 µW (for a current of 60 µA), which is below the limit of a system with these electrodes, and was therefore selected for hydrogen generation.

Supplemental Note #4 Influence of the mass-transport on electrodes and biofuel cell characteristics.

Although the polarization and power curves present similar values for two or one-compartment experiments (Fig. 3c and d in the main text), the anode is limiting in the first case while it is the cathode in the second case. The curves appear noisier for the two-compartment experiment due to a higher sensitivity of cathodes to mass-transport fluctuations. To illustrate it, cyclic voltammogramms in the presence of glucose and oxygen with (thick lines in Supplementary Figure 5a) or without (thin lines) active O_2-bubbling are compared. While the anode loses some 25 % of its current in the absence of agitation, the cathode is much more influenced by mass-transport with a loss of almost 80 %, changing from a current of -2.4 mA to around -0.5 mA. At the same time, the noise, caused by variations of oxygen transport through buffer agitation by the O_2-bubbling, disappears and the cathodic current becomes smoother. Supplementary Figure 5b shows the impact of the cathode behavior, which is the current limiting electrode of the biofuel cell, on the polarization curve. The current is measured during polarization at 5mV s^{-1} under similar conditions of agitation as for Supplementary Figure 5a. The noisy signal disappears when the solution becomes quiescent, and the short-circuit current follows the trend of the cathode measured independently, with around -2.2 mA under active O_2-bubbling and -0.45 mA in quiescent buffer.

Supplemental Note #5 Electrical measurements.

Current and voltage characteristics were measured as a function of time for the different elements of the electrical scheme of the set-up. In the two-compartment set-up, 185µW is the power consumed to perform electrolysis for a 50µm-diameter platinum electrode in phosphate buffer 100mM at 25°C for an imposed tension of 3.1V, with a corresponding current of about 60µA. In this case the electrolysis is powered by the BFC using only the boost-converter on the demo board, which delivers a stabilized tension of 3.1V as long as it is able to gather enough power from the BFC. However electrolysis with a working electrode of this size can also occur at lower potentials (see Supplementary Figure 4b above), obviously leading to smaller currents. This is the case for the one compartment set-up for which at 37°C the electrolyser operates typically at 2.9V with a current of around 40µA flowing through it, corresponding *in fine* to a power of 115 µW. Supplementary Figure 6a shows the voltage evolution at the input (red line) and output (black line) of the boost-converter, which respectively correspond to the BFC output and flyback input.

During more than 80 minutes, the boost-converter receives enough power from the BFC to maintain its 3.1 V output voltage. Typical power values of 250, 175 and 115 µW at the in- and output of the boost-converter as well as the electrolyser input (flyback output) were measured during the 80 minutes of optimal functioning of the system, resulting in power efficiencies of 70 and 65 % for the boost-converter and the flyback, respectively. These values are calculated as follows:

The Flyback was designed to operate in the discontinuous mode (DCM) with the aim of limiting the volume of the system, implying the reduction of the transformer inductance. Another reason to use the DCM mode is the possibility to obtain a more regular flow of dihydrogen at the electrolyzer because for constant input voltage and switching parameters,

the Flyback output power remains almost constant in a reasonable range of load impedances (see example below).

Therefore, for our application we have chosen a transformer with a magnetizing inductance L_{ms}= 0.01H and adjusted switching to run the Flyback in the discontinuous mode with a voltage gain near 1. The operating point of our Flyback is indicated as a circle on the Supplementary Figure 7, which illustrates the delimitation of the CCM/DCM boundary [1].

To calculate this point we have chosen the load resistance R_L=55kΩ as an example (close to the ones observed during our experiments) and a value of the duty cycle (α) of 0.056 adjusted on the TS3003DB (αT = 6.27µs, T=1/f_s = 111.11µs, see Supplementary Note 2). It can be seen that the Flyback is functioning in the DCM mode, which significantly changes its properties as compared to the continuous conduction mode (CCM). It has to be noticed that this transformer has been chosen (Murata 78601/9C) for its volt-time product, compatible with its peak currents (about 2mA, see below) in order to avoid magnetic saturation.

The transfer function in the lossless case then becomes for the DCM mode (formula 5.153 p215 in [1]):

$$M_{V\ DC(lossless)} \equiv \frac{V_o}{V_I} = \frac{D}{n}\sqrt{\frac{R_L}{2f_s L_{ms}}} \qquad 1$$

where n, the turn ratio, is 1 in our transformer .

Or alternatively, in the case of a lossy Flyback (equation 5.194 p 221 in [1]):

$$M_{V\ DC} = \sqrt{\eta} M_{V\ DC(lossless)} = \frac{D}{n}\sqrt{\frac{\eta R_L}{2f_s L_{ms}}} \qquad 2$$

where η is the efficiency.

In contrast to the CCM mode, the transfer function in the DCM mode depends on the load of the Flyback, and is proportional to the square root of it.

Supplementary Figure 8 shows with black dots the transfer function measured for the complete Flyback, fed by a 3.1V DC power supply and feeding load resistances (resistors) ranging from 1 to 100kΩ .

$\frac{V_o}{V_I}$ evolves linearly with $\sqrt{R_L}$, demonstrating that the Flyback under these conditions works in DCM mode.

In the same experiment the input current measurement also allows to check that the effective value of inductance in the electronic circuit, in its conditions of use, is close to 10mH. Inductance values, which are sensitive to environmental conditions such as temperature, but also to the excitation signal, can indeed divert from the manufacturer's nominal value.

The input power at the primary winding of the Flyback follows the expression 5.162 of [1]:

$$P_I = \frac{D^2}{2f_s L_m} V_I^2 \qquad 3$$

From this the inductance can be estimated:

$$L_m = \frac{D^2}{2f_s} \frac{V_I}{I_I} \qquad 4$$

With V_I=3.1V and I_I=54,4 ±1.4µA in the primary winding this gives a L_m value between 10.0 and 10.2mH.

Red dots depict the theoretical voltage transfer defined from the equation in the lossless case given above for 10mH. The ratio between experimental and theoretical points allows to visualize the efficiency of the Flyback, as the transfer functions of a theoretical lossless and a practical lossy Flyback differs by a factor $\sqrt{\eta}$. The small blue points indicate the data of a typical experiment for the one-compartment production of H_2 powered by the BFC and described in the manuscript. In the first period of 80 minutes, where the Flyback is supplied by a tension of 3.1V provided by the boost-converter, the resistance of the electrolyser, which represents the load resistance of the Flyback, increases slowly from 60 to

80 kΩ, because of platinum poisoning. One can note the good correspondence between output/input voltage ratios obtained with linear loads and with the more complex electrolyser. These typical values of resistance then ensure low variation of output voltage and efficiency during an experiment.

A capacitor (C=47nF) with low ESR was experimentally selected for an acceptable ripple voltage. For calculating the losses in the diode, one needs to know at least five parameters:

- the maximum current in the diode (1,9 mA typical computed according to formula 5.180 in [1]) to estimate the upper limit of the forward threshold voltage.
- this forward threshold voltage is the intercept at the voltage axis of the diode I=f(V) curve tangent at the maximum current ordinate. A value of 250 mV is obtained from the BAT42WS Vishay's datasheet after linear transposition.
- the average current (which is also the flyback average output current, has a typical value of 40 µA in one-compartment experiments).
- the rms current $I_{D\,rms}$which has a value of around 260µA, determined from formula 5.181 in[1].
- the dynamic resistance of the diode around its working point. We get this value by estimating the reciprocal of the tangent slope of curve I =f(V) at $I_{D\,rms}$ ordinate and find 106 ohms.

The BAT42WS diode was also chosen because of its low reverse current of around 0.1µA in our range of voltage.

Total resulting losses in that diode were estimated using adequate formulas (5.182 and 5.184, 5.185) in [1], or the ST application note [2], and do not exceed 13 % of total output power. When considering a global Flyback efficiency of 0.65 (see below) the diode losses represent only 8.5 % of input power, which is very reasonable.

The formula 5.189 of page 221 of [1] allows one to determine a maximal theoretical limit for the efficiency and permits one to evaluate whether the 0.65 efficiency measured during one-pot experiments is realistic with this Flyback. All component parameters were directly taken or evaluated from datasheets, or eventually evaluated from Capitaine et al [3] where a Flyback based on the same or very similar components than the present ones is analyzed in terms of efficiency. We obtain a maximal theoretical efficiency of 0.86 which seems to be close to the one found by Capitaine et al for a system also working in discontinuous mode and for an input power of 90µW. [3]

The practical efficiency of the present Flyback for different loads is plotted below, derived from the experiments related to the preceding graph.

One can see that the efficiency is very stable at around 0.65 for the higher values of load resistances (resistors) including also resistances (small blue points) of the electrolyser deduced from the one-compartment H_2 production experiment (see also Supplementary Figure 6b).

This efficiency stability with respect to the input voltage of the Flyback loaded with the electrolyser, and fed by a DC power supply, was also verified: the relative decrease in power efficiency was less than 5% for an input voltage down to 2.2V. The obtained optimal experimental power efficiency of 0.65 is moreover very similar to the 0.68 that has also been determined by Capitaine et al (Fig. 5 in [3]).

Other examples of Flyback systems, including diodes, developed with the objective to harvest energy from Microbial Fuel Cell, reported equivalent efficiencies. Khaled et al [4] reported a 0.712 efficiency when harvesting energy from a MFC of 755µW maximum power. Capitaine et al. get, after further development of their system, an efficiency of 0.75 for a 30µW input.[5]

The experimental values reported above consider the Flyback as one part of our electronic system with an efficiency of 0.65 as illustrated by Supplementary Figure 9. However, our system also includes the boost-converter as a low input voltage energy harvester. It has an efficiency varying between 0.70 and 0.73 (see Fig. 2,6,7 of the BQ25504 datasheet) [6]. Combining these two values leads to a total experimental efficiency of 0.46-0.48. Please notice that a system similar to the present one, with a BQ25504 boost-converter connected to a flyback for energy harvesting from a microbial fuel cell, reported a similar "extracted energy" of "about 50% of the original available energy in the MFC" [7]. Therefore, the findings reported in the present work are in very good agreement with literature results.

These efficiencies values remained constant in spite of the general decrease in power that occurred after the initial 80min period. Indeed, due to the power evolution of the biofuel cell as a function of time, corresponding to a decrease of the OCP concomitant with a decrease in peak power voltage and maximum power, the input voltage of the boost-converter has to lower in order to provide enough power to maintain the 3.1 V. Once the maximum power that the BFC can supply goes below 250 µW, the voltage at the boost-converter output starts to decrease due to a lack of input power (Supplementary Figure 6b).

Supplementary Methods.

Electrolysis cell

An experimental cell was designed with planar parallel faces to improve the observation of the gas microbubbles (Supplementary Figure 10a). 75 cm² and 25 cm² culture flasks from Nunc were cut at their top and 3mm-diameter holes were drilled in their center at the bottom. 3 mL syringe bodies were inserted in holes created in the sides walls of the bigger flask to ultimately constitute the in- and outlet of thermoregulated water. A 1 mm hole was punched in the center of 1 cm² pieces of PDMS with 1 mm thickness (Bayer Silicones). They were fixed respectively at the inside (for the smaller flask) and the outside (for the bigger one) of the 3 mm-holes with Araldite glue. The two flasks were aligned by passing a microelectrode through the PDMS holes and stabilized with silicone paste (CAF4 from Bluestar Silicon). This results in a tight and waterproof entrance for inserting the microelectrode in the experimental cell.

H_2 collection and measurement

An engineered plane needle, a glass capillary (ringcaps, Hirschmann Laborgeräte, reference 9600150) and a shortened Pasteur pipette were connected with 1 cm-pieces of Tygon® tubing (Saint-Gobain) and glued with epoxy. A thin plastic rod covered with millimeter grading paper was positioned parallel to the capillary at the level of the connection tubing (Supplementary Figure 10b).

Just before the experiment, the collector was rendered hydrophilic by 1 h treatment with fresh piranha (85/15 % v/v H_2SO_4 98 %/ H_2O_2 35 % v/v) with regular replacement of the solution in the capillary by syringe aspiration. It was then rinsed ten times with milli-Q water. The collector was then fixed over the electrolysis solution maintained by the syringe, filled with the buffer, and a 5 µL air bubble was introduced inside the capillary. The collector was placed over the microelectrode with a 1-2 mm distance from the bottom of the cell to avoid the possible introduction of O_2 bubbles from the oxygen supply.

For H_2 volume measurement, the gas volume in the capillary is determined from the pictures that are regularly and automatically recorded (Supplementary Figure 10c). It allows to calculate the volume of gas by simple conversion of the capillary length into volume. The measurement precision of 0.5 mm in length corresponds to 0.41 µL. These volumes can be compared to the calculated volumes V_c (in µL) originating from the charge C (in µC) passing through the electrolyser, following the formula:

$$V_c = -\alpha \frac{C}{nF} \times V_m \qquad 5$$

where n = 2 is the number of electrons necessary for the formation of one molecule of H_2, the minus sign denotes the negative value of reduction currents, F = 96485 C mol^{-1} the Faraday constant, V_m = 24.4L mol^{-1} is the molar volume of a perfect gas at 298 K and α = 310/298 is the correcting factor for the experimental conditions of temperature (37 °C).

Supplementary References

1. Kazimierczuk, M. K. *Pulse-width Modulated DC–DC Power Converters*. (John Wiley & Sons, 2008).
2. Calculation of conduction losses in a power rectifier. *ST Appl. Note* (2011).
3. Capitaine, A. *et al.* Loss analysis of flyback in discontinuous conduction mode for sub-mW harvesting systems. in 1–4 (IEEE, 2016). doi:10.1109/NEWCAS.2016.7604810
4. Khaled, F., Allard, B., Ondel, O. & Vollaire, C. Autonomous flyback converter for energy harvesting from microbial fuel cells. *Energy Harvest. Syst.* **3,** 153–160 (2016).
5. Capitaine, A., Pillonnet, G., Chailloux, T., Ondel, O. & Allard, B. 10 μW converter for energy harvesting from sedimentary microbial fuel cells. in 337–340 (IEEE, 2017). doi:10.1109/MWSCAS.2017.8052929
6. Texas Instruments. BQ25504 Ultra low-power boost-converter with battery management for energy harvester applications, SLUSAHOC. (2015).
7. Khaled, F., Ondel, O. & Allard, B. Microbial fuel cells as power supply of a low-power temperature sensor. *J. Power Sources* **306,** 354–360 (2016).
