# Supplementary material for: Uphill production of dihydrogen by enzymatic oxidation of glucose without an external energy source
Source: Nat Commun. 2018 Aug 13;9:3229. doi: 10.1038/s41467-018-05704-5 (PMC6089969; doi:10.1038/s41467-018-05704-5)
Supplement: Supplementary file 2 — Description of Additional Supplementary Files [file 41467_2018_5704_MOESM2_ESM.pdf]

Description of Additional Supplementary Files

File Name: Supplementary Movie 1
Description: Production of dihydrogen at a platinum microelectrode by water electrolysis, powered by a glucose/O_2 biofuel cell (BFC), in a single compartment cell.

File Name: Supplementary Movie 2
Description: Zoom-in of the microelectrode showing the hydrogen production.

From t = 0s to t = 20s : BFC connected; electrolyser connected
At t = 20s : BFC disconnected; electrolyser connected
At t = 28s : BFC connected; electrolyser disconnected
At t = 37s : BFC connected; electrolyser connected
